# Integrated Analysis of Brain Transcriptome Reveals Convergent Molecular Pathways in Autism Spectrum Disorder

**DOI:** 10.3389/fpsyt.2019.00706

**Published:** 2019-10-08

**Authors:** Xiaodan Li, Yuncong Zhang, Luxi Wang, Yunqing Lin, Zhaomin Gao, Xiaolei Zhan, Yan Huang, Caihong Sun, Dong Wang, Shuang Liang, Lijie Wu

**Affiliations:** ^1^Department of Child and Adolescent Health, School of Public Health, Harbin Medical University, Harbin, China; ^2^Province Key Laboratory of Children Development and Genetic Research, Heilongjiang, China; ^3^College of Bioinformatics Science and Technology, Harbin Medical University, Harbin, China; ^4^Department of Bioinformatics, School of Basic Medical Science, Southern Medical University, Guangzhou, China

**Keywords:** autism spectrum disorder, corpus callosum, prefrontal cortex, protein interaction network, WGCNA

## Abstract

Autism spectrum disorder (ASD) is a set of complex neurodevelopmental disorders with etiology that remains elusive. Although there is a mounting body of investigation in different brain regions related to ASD, our knowledge about the common and distinct perturb condition between them is at the threshold of accumulation. In this study, based on protein–protein interactions, post-mortem transcriptome analysis was performed with corpus callosum (CC) and prefrontal cortex (PFC) samples from ASD individuals and controls. Co-expression network analysis revealed that a total of seven (four for CC set, three for PFC set) core dysfunctional modules strongly enriched for known ASD-risk genes. Three quarters of them in CC set (M4, M6, M29) significantly enriched for genes annotated by genetically associated variants in our previous whole genome sequencing data. We further determined transcriptional and post-transcriptional regulation subnetwork for each ASD-correlated module, including 47 pivot transcription factors, 130 pivot miRNAs, and 7 pivot lncRNAs. Moreover, there were significantly more interactions between CC-M4, -M6, and PFC-M2, mainly involved in synaptic functions and neuronal development. Our integrated multifactor analysis of ASD brain transcriptome profile illustrated underlying common and distinct molecular mechanisms and the module crosstalk between CC and PFC, helping to shed light on the molecular neuropathological underlying ASD.

## Introduction

Autism spectrum disorder (ASD) is a group of neurodevelopmental disorders characterized by high degree of clinical and genetic heterogeneity. The Diagnostic and Statistical Manual of Mental Disorders-Fifth Edition (DSM-5) defines ASD by deficits in social communication and interactions, as well as by repetitive behaviors and restrictive interests, with onset in early development. Currently, 1 of every 59 children in the United States is diagnosed with ASD (1), whereas the prevalence of ASD in China is 8.3 per 10,000, which is likely underestimated due to the strict diagnosis criteria (2). Consistent with the primary features of ASD, many studies demonstrated the association between brain abnormalities and the disease, yet no clear and common molecular or pathology mechanisms have proved to be responsible for this disease so far. Animal experiments showed that abnormal brain regions were mainly involved in the temporal lobe, cerebellar cortex, frontal lobe, hypothalamus, and the striatum by 26 different ASD mouse models (3). Postmortem and structural magnetic resonance imaging studies have highlighted the frontal lobes, amygdala and cerebellum as pathological area in ASD (4). Although several genetic and environmental risk factors have been identified, we do not have a firm understanding of the pathophysiological basis of this complex disease by now.

Among the human brain regions implicated in the pathophysiology of ASD, the prefrontal cortex (PFC), as the center of the highest-order cognitive functions, has always been focused on due to its role in the cognitive, decision-making and social behavior (5). Previous studies have confirmed that some microglial activation in ASD was associated with a neuron-specific reaction in the dorsolateral prefrontal cortex (6). The corpus callosum (CC), predominantly populated by oligodendrocyte cells, has been the largest white matter tract in the human brain, interconnecting homologous association areas of both hemispheres (7). Dysplasia of the CC often involves in social impairments similar to those seen in high-function people with ASD, encompassing diminished social self-awareness, difficulty in imaging the social perspective of others, poor conversation skills and restricted verbal expression of emotional experience (8). Though its contribution to cognition and behavior remains unclear, the indirect relationship between the volume of CC in anatomy and ASD severity suggested its susceptibility of etiology (9).

Furthermore, brain function is governed by precise regulation of gene expression across its anatomically distinct structures. However, the regulation programs of gene expression in human are controlled by thousands of transcription factors (TFs), cofactors and chromatin regulators, involving transcriptional and post-transcriptional levels. Dysregulation of these programs may cause a broad range of diseases (2, 10). Transcription Factor 4 (TCF4) binding sites were found in a large number of neuronal genes that were implicated as genetic risk factors for common neurodevelopmental disorders (11). Previous studies found miR-146a up-regulation was the most common miRNA dysregulation event in neurodevelopmental disorders such as ASD, epilepsy, and intellectual disability (12). Long non-coding RNA (lncRNA) Shank2-AS was abnormally expressed in patients with ASD and might affect the structure and growth of neurons by regulating its sense strand gene *Shank2* expression (13).

We reasoned that distinct pathogenic mechanisms in CC and PFC area in ASD might converge on common pathways that are not yet well understood. Since systems biology made it possible to study larger and more intricate systems than before, gene set and network-based analysis became powerful tools for evaluating putative genetic risk factors and dysfunctional modules for diseases. RNA-sequencing data of CC (GSE62098) (14) have provided DEGs between ASD children and typically developing controls and was used to confirm the involvement of a protein interaction module (with GO enrichment for synaptic transmission) in ASD. Li et al. (14) examined the expression specificity of the module in the CC with immunochemical method and showed that the human CC was predominantly populated by oligodendrocyte cells. Then multiple genomic data further revealed a significant involvement of this module in the development of oligodendrocyte cells in mouse brain. For PFC data (GSE102741), Wright et al. (15) revealed that seven histamine genes (*SNORA74A*, *SNORA53*, *SNORD17*, *SNORA54*, *SNORA74B*, *SNORD114-23*, and *RP6-206I17.3*) showed altered expressions in ASD children, suggesting that the histaminergic system might also be altered in ASD. However, few efforts have been made to conduct a comprehensive approach to decipher tissue specific pathogenic mechanism of ASD.

To this end, weighted gene co-expression network analysis (WGCNA) was performed in this paper based on integrated RNA-seq data, protein–protein interactions (PPIs) and TF-, ncRNA-target interactions. In our previous study, we have conducted a comprehensive scan of genomic variance differences among three pairs of monozygotic twins with whole genome sequencing (WGS) (16). Here we extend our genomic analysis to the transcriptome profile of ASD-affected individuals aimed at identifying common and distinct transcriptional alterations in dysfunctional modules of CC and PFC, which may prove to be a crucial step to better understand ASD.

## Materials and Methods

### Data Resources and Differential Expression (DE) Analysis

Gene expression profile data of 24 corpus callosum samples [GSE62098 (14), 12 ASDs, 12 controls] and 52 prefrontal cortex samples [GSE102741 (15), 13 ASDs, 39 controls] were downloaded from the NCBI Gene Expression Omnibus (GEO) (17) database. For GSE62098, original samples were requested from Autism Speak’s Autism Tissue Program (www.atpportal.org) and NICHD Brain and Tissue Bank (http://medschool.umaryland.edu/btbank/). For GSE102741, samples were collected at National Institute of Mental Health brain collection (18), the University of Maryland Brain and Tissue Bank (www.medschool.umaryland.edu/btbank/) and the Stanley Medical Research Institute sample characterization (www.stanleyresearch.org). Subjects with evidence of drug use, alcohol abuse or psychiatric illness were excluded from the control cohort. Details of clinical characterization, neuropathological examinations and toxicological analyses are available on their respective websites.

Two sets of data were processed separately. The quality control was performed by Fast-QC (version 0.11.5). Then, filtered reads were used to map to the hg38 genome reference genome (GRCH38) using HISAT2 (version 2.1.0) (19) with default parameters. Fragments Per Kilobase transcriptome per Million (FPKM) reads values and raw counts of gene expression were calculated within StringTie (20) (version 1.3.3). Based on raw counts tables, differentially expressed genes (DEGs) were ranked and filtered by DESeq2 (R package) (21) for further analysis (|Fold Change| > 1.2, *p*-value < 0.05).

### Gene Co-Expression Network Analysis

WGCNA was applied to derive gene networks based on all pair-wise gene expression correlations between genes (22). A human protein–protein physical interaction (PPI) subnetwork (combined score ≥ 900) was firstly extracted from STRING database (v10.5) (23), consisting of 9,635 proteins and 170,876 interactions. Then, DEGs and their interactors in the PPI network were identified to construct weighted gene correlation network. Finally, the expression profile of genes in the weighted gene correlation network was input to WGCNA for co-expression modules. For GSE62098 (CC set), a power of 12 was chosen in this study and the parameters minModuleSize = 20, minimum height = 0.15 were set to cut tree. For GSE102741 (PFC set), power = 8, minModuleSize = 20, minimum height = 0.15 were set to cut tree. For each co-expression module, nodes with KME value > 0.8 were considered as hub genes.

### Identification of Pivotal Regulators

TF-target interactions (785 TFs, 2,489 target genes) were recruited from AnimalTFDB v3 (24) and TRRUST v2 (25). For post-transcriptional regulation, miRNA- (1179 miRNAs, 6,677 target genes) and lncRNA-target interactions (93 lncRNAs, 7,744 target genes) were recruited from RAID v2.0 (26). If a factor (i) has >1 interactions and (ii) the number of its target nodes significantly enriched for the module (hypergeometric test, *p* < 0.05) (27), the factor was identified as a pivot regulator.

### Identification of Crosstalk Module Pairs

First, we compiled a list of 3,352 protein coding genes differently expressed between CC (white matter) and PFC (frontal lobe) using the Allen Human Brain Atlas (28) (|Fold Change| ≥4, *p*-value < 0.05), 814 were on STRING PPI network. Tissue-specific modules were defined as modules of which specific expression parts containing DEGs. We counted the number of interactions between each tissue-specific module. Then by keeping the number of nodes unchanged compared to the corresponding tissue-specific module genes on PPI network, paired gene sets were randomly sampled for 100,000 times and also counted the number of interactions of each pair, which allowed us to assign the *p* value. Significant crosstalk module pair was defined as a pair of gene sets from CC and PFC set separately which of significantly more interactions between each other than random pairs (permutation test, *p* < 0.05).

### Functional Enrichment Analysis

Gene ontology biological processes (GO-BP), Kyoto Encyclopedia of Genes and Genomes (KEGG) enrichment analysis were carried out with R package clusterProfiler (29). For pivot miRNAs, functional annotations were performed on DIANA-miRPath v3 (30).

## Results

### Altered Gene Expression and Weighted Gene Co-Expression Network Analysis (WGCNA)

**Figure 1:**
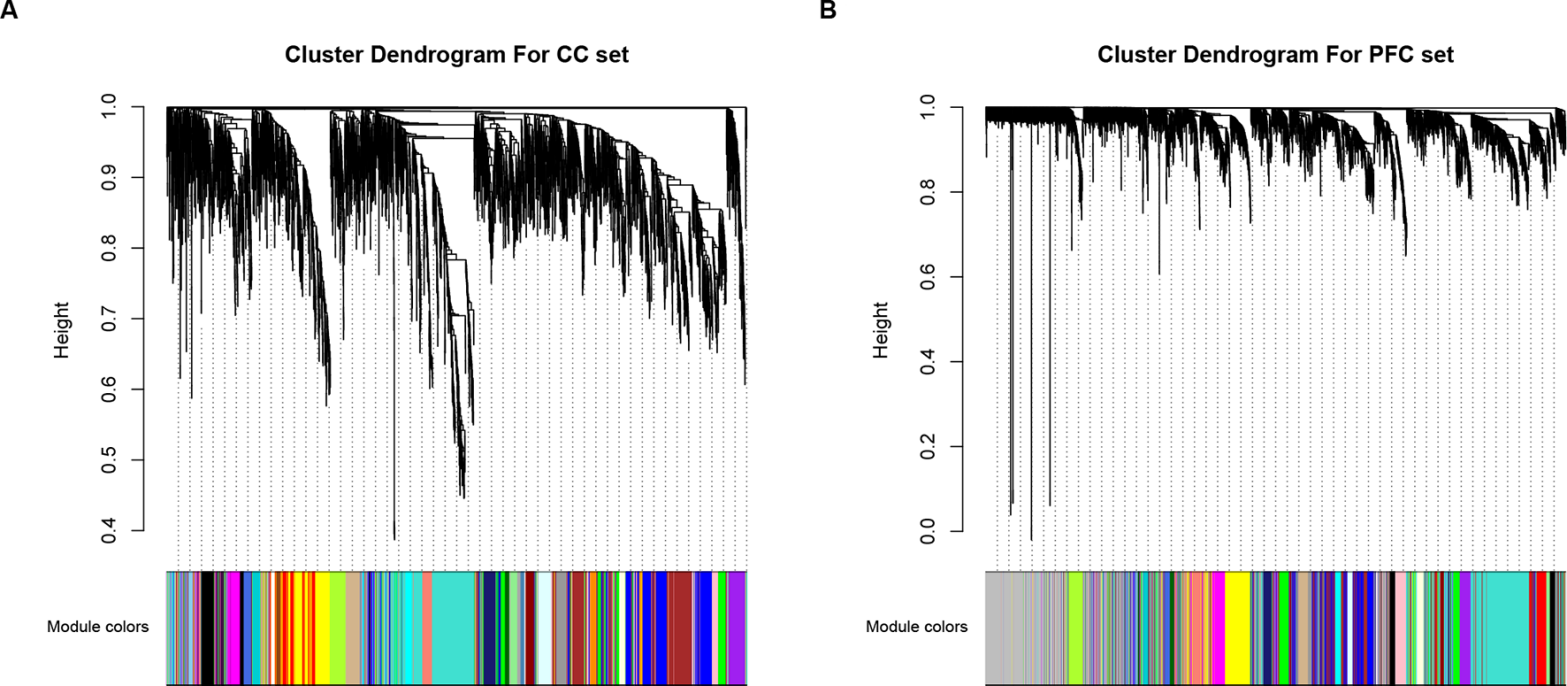
Visualization of WGCNA results. Clustering dendrograms of genes within **(A)** CC and **(B)** PFC subset, X-axis represents genes and Y-axis represents height of the gene tree. Total of 30 (CC set) and 28 (PFC set) co-expression modules corresponding to different color bars while grey bars represent genes not included in any co-expression module.

We performed a differential expression (DE) analysis to determine the gene significantly expressed in ASD compared to controls, and a total of 653 and 720 DEGs were identified for further analysis, in CC and PFC data respectively (|Fold change| > 1.2 and *p*-value < 0.05, **Supplementary Table S1**). Since interacted genes imply co-expression (31), human protein–protein interaction (PPI) subnetwork based on DEGs containing 3,492 interactors for CC set and 3,243 for PFC set were constructed for additional analysis (see *Materials and Methods* section). Then based on WGCNA, 30 (CC set) and 28 (PFC set) dysfunctional modules were initially determined (**Figure 1**). Taking the effectiveness of the modules into account, modules containing DEGs and size less than 500 genes were kept for further analysis (**Supplementary Table S2**).

### Dysfunctional Co-Expression Modules in CC and PFC

In order to detect whether there were common or distinct contributors perturbed in the functional modules, remained modules were further filtered by genomic variation burden and correlation with ASD. Based on our previous study (16), discordant variations in monozygotic twin (DVMT) including single nucleotide variants (SNVs), small insertions and deletions (InDels), and copy number variations (CNVs) presented in at least two twin pairs were filtered as putative ASD risk sites. A subset of 1,714 protein coding genes annotated by three types of DVMT were used to determine the genomic variance burden in the present study and made further analysis. First, we calculated the number of genes involved in ASD susceptibility to determine whether any of co-expression modules were associated with ASD, using SFARI Gene list (https://gene.sfari.org/autdb/). M4, M6, M17, and M29 for CC set and M2, M6, and M8 for PFC set showed significant enrichment, which were referred to be dysfunctional modules (*p* < 0.05). Next, we confirmed the results above by calculating overlaps between module nodes and DVMT genes using our previous WGS data, which were significantly converged into M4, M6, M7, M9, M29 (CC set), and M5, M11, M24 (PFC set) (*p* < 0.05). Notably, most of ASD-associated modules (M4, M6, and M29) of CC set showed DVMT significance, while none of that was observed with PFC set (**Table 1**, **Supplementary Table S3**).

**Table 1:** Co-expression modules significantly enriched for SFARI and/or DVMT genes.

Module	DEGs/Module size	SFARI	*p*-value (a)	DVMT	*p*-value (b)
CC-4*	13/281	40	**6.26E-08**	35	**4.75E-04**
CC-6*	9/168	20	**1.71E-03**	19	**2.49E-02**
CC-7	1/167	10	6.02E-01	21	**5.91E-03**
CC-9	8/142	8	6.71E-01	17	**2.00E-02**
CC-17	2/75	9	**3.01E-02**	9	7.76E-02
CC-29*	3/32	5	**3.67E-02**	7	**5.73E-03**
PFC-2	58/315	37	**4.26E-04**	19	7.78E-01
PFC-5	11/179	17	9.35E-02	20	**1.07E-02**
PFC-6	21/152	23	**1.63E-04**	15	1.01E-01
PFC-8	15/147	19	**4.12E-03**	15	8.13E-02
PFC-11	56/112	2	9.97E-01	13	**4.38E-02**
PFC-24	2/24	3	2.19E-01	9	**1.65E-05**

*Denotes for modules that were significantly enriched for both ASD and DVMT genes, p-value (a) and (b) represent enrichment results for SFARI and DVMT genes respectively, p-values in bold denote which statistical threshold has been reached (<0.05).

**Figure 2:**
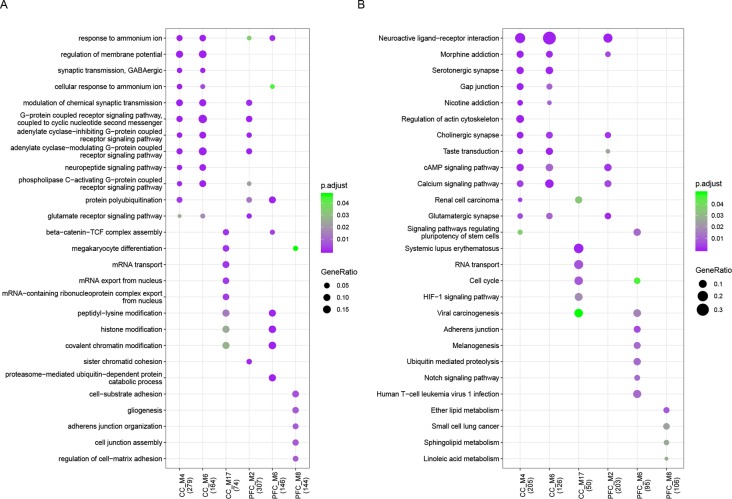
Functional enrichment results. **(A)** GO biological process and **(B)** KEGG pathway enrichment results of ASD-related dysfunctional modules which enriched for at least one GO term or pathway with *p*.adjust < 0.05. The node size represents gene ratio and color represents *p*.adjust value.

Functional enrichment analysis was performed for each dysfunctional module and revealed multiple biological processes of gene ontology (GO-BP) critical to major functions for both sets (**Figure 2**, **Supplementary Table S4**), including cognition, learning or memory, long term depression, nervous system development, and synaptic function. KEGG pathway analysis showed that common pathways like neuroactive ligand–receptor interaction, calcium signaling, MAPK and PI3K-Akt signaling were observed. We found that 28% (20/72) and 29% (9/31) of genes involved in neuroactive ligand–receptor interaction, and in CC and PFC set respectively, have been reported to be related to ASD. There were also module-specific terms like RNA transport and localization involved in M17 of CC set, protein ubiquitination and regulation of cell-substrate adhesion in M6 and M8 of PFC set, respectively. The ubiquitin–proteasome system has been considered to be a major non-lysosomal proteolytic process that regulates the levels of cellular proteins related to synaptic plasticity and long-term memory, and to ASD (32, 33).

Hub genes are those that show most connections in the network and of great use in identifying genes with significant biologically meaning. In line with functional enrichment results, *CACNA1C*, one of the hubs of CC M6 presented in both SFARI and WGS set, in which genetic variation have been associated with ASD, Major Depressive Disorder, Schizophrenia as well as some undiagnosable psychiatric illness (34). Other hub genes (*GABBR2*, *GRM7*, *MEF2C*, *SCN2A*, *KMT2C*, and *DAGLA*) dysfunction have been reported to contribute to ASD and other psychiatric disorders such as Attention-Deficit Hyperactivity Disorder (ADHD) (35), Huntington’s disease (36), and Rett-syndrome (37). These observations supported the existence of shared dysfunction convergence but might distinct mechanism under CC and PFC in ASD.

### Pivot Regulators of Dysfunctional Modules

**Figure 3:**
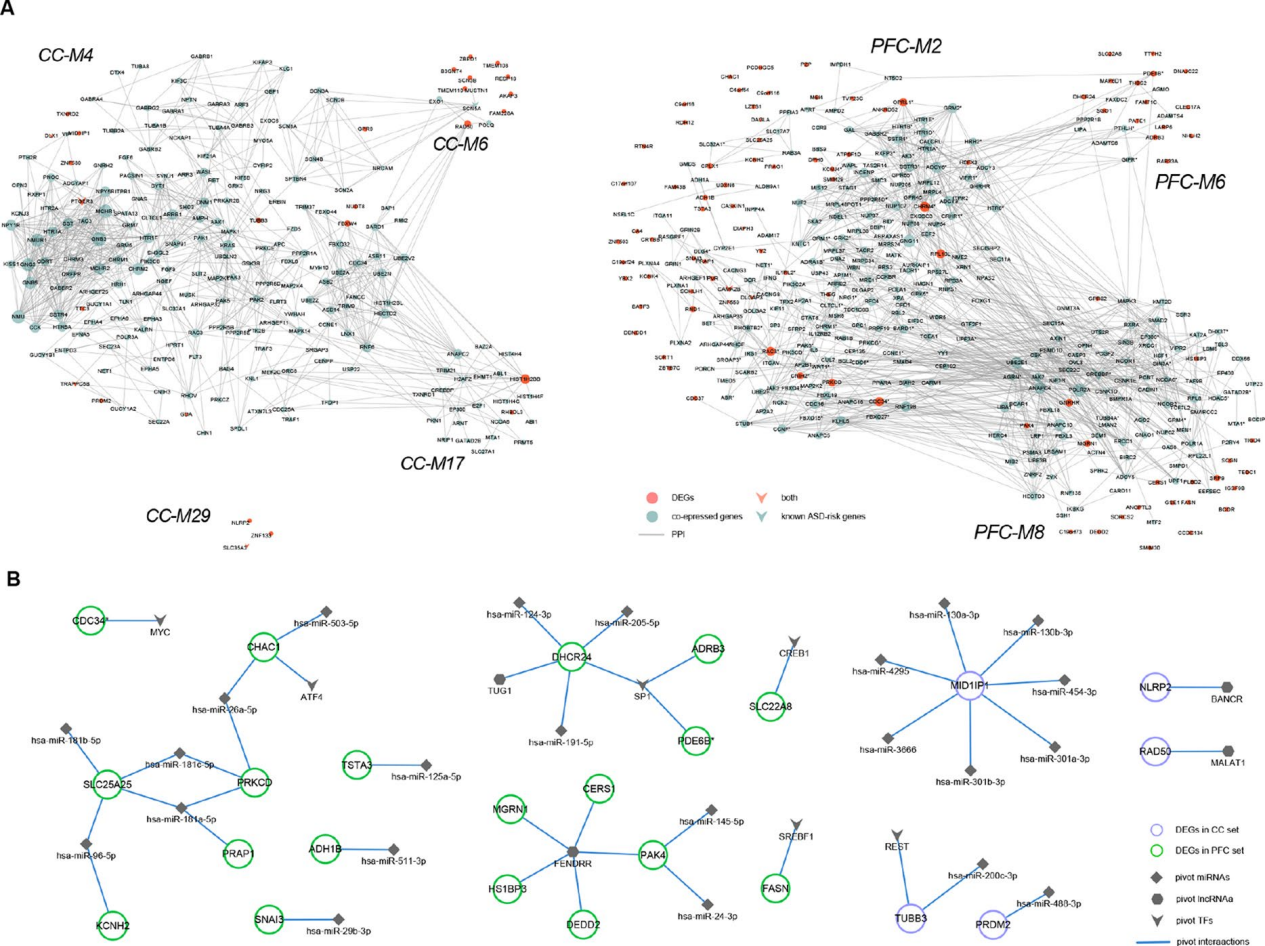
Regulatory subnetworks. **(A)** DEGs and their direct or indirect interactors within ASD-related modules (CC-M4, -M6, -M17, M29, and PFC-M2, -M6, -M8). The node size is proportional to degree. **(B)** The transcriptional and post-transcriptional pivot regulator-DEG subnetworks of ASD-related modules, containing 6 pivot TFs, 4 pivot lncRNAs, and 23 pivot miRNAs.

We determined pivotal regulators by transcriptional and post-transcriptional level regulations among modules significantly enriched for genomic variants and/or known ASD-risk genes (see *Materials and Methods* section, **Supplementary Table S5**). Then, regulator-DEG and DEG-interactor subnetworks were constructed (**Figure 3**).

Recent whole exome sequencing (WES) has revealed substantial overlap in ASD-risk genes and cancer (38). The transcription factor MYC, regulating both CC-M4 and PFC-M2, is a cell growth regulator which strongly oncogenic, and estimated to contribute to most cancers (39). Transcription factor SP1, regulating CC-M6, -M7, and PFC-M5, -M8, has been reported dysfunctional in the anterior cingulate gyrus (ACG) of ASD brain and the potential ASD candidate gene *PTEN* (in PFC-M5) regulated by SP1 was shown to be over-expressed (40, 41). CREB1, down-regulated at PFC, can regulate CC-M4, -M9, and PFC-M8, assumed to be linked to cognition and behavior related to butyrate dysregulation in ASD (42). Moreover, *de novo* deletion within CREB1 was observed in a girl with developmental delay, autistic traits and Rett-like features (43). Pivot TFs E2F1, regulating CC-M17 while down-regulated at PFC, was involved in cell cycle regulation and apoptosis as well as functionally related to obesity which was consistent with the function enrichment results of M17 (44).

LncRNAs are more abundant in the human brain and are involved in neurodevelopment and neurodevelopmental disorders, including ASD (45). It was observed that most pivotal lncRNAs examined in this study have been reported related to neurodegenerative disease and cancers. *TUG1*, regulating M7 of CC set and M2, M5, and M8 of PFC set, was reported to be a tumor suppressor in breast cancer and high correlations were found between ASD prevalence and the incidence of *in situ* breast cancer (46, 47). *FENDRR*, regulating CC M4, M7, and PFC M6, is a kind of endothelial gene critical for vascular development that could inhibit breast cancer cell proliferation (48). Moreover, *UCA1*, regulating CC M6, M17, and PFC M6, has been identified as a pivotal regulator in the tumorigenesis of glioma which represent the most common solid tumor of childhood (49, 50).

MicroRNAs are post-transcriptional regulators that play key roles in brain development, synapse formation and fine-tuning of genes underlying synaptic plasticity and memory formation (51). We perform GO enrichment analysis and found that pivot miRNAs regulated DEGs were significantly enriched for GO terms like cellular protein modification process, neurotrophin TRK receptor signaling pathway, nervous system development, and axon guidance. Furthermore, KEGG pathway analysis showed that both sets were convergent into pathways like ErbB signaling, long-term depression, Estrogen signaling pathway and so on, which were closely related to ASD (52, 53). Taken all together, these results indicated that pivot regulators might play an important role in the disease process directly or indirectly.

### Module Crosstalk Between CC and PFC

Modules do not act alone. We further analyzed the module interactions between CC and PFC set (**Figure S1**). To this end, CC-M4, -M6, and PFC-M2 were determined as tissue-specific modules using the Allen Human Brain Atlas and module pairs PFC M2-CC M4, and PFC M2-CC M6 were found to be connected with each other significantly with more interactions than random gene set pairs (*p* = 5.00E–05 and 2.50E–04, respectively) (see *Materials and Methods* section). All of these three modules were most significantly enriched for “neuroactive ligand–receptor interaction” pathway and participated in neuro system development, indicating the association of ASD pathogenic mechanism between CC and PFC.

## Discussion

Although the symptoms of ASD are the most striking among the behavioral and functional manifestations of affected individuals, however, findings are profoundly heterogeneous and the etiology of ASD is still unclear. The corpus callosum plays a central role in mediating signal communication between the brain hemispheres through the axons extending from different cortical layers (54), whereas the prefrontal cortex plays an essential role in the organization and control of goal-directed thought and behavior (55). Recent advances have reported ASD clinical heterogeneous based on typical brain functional regions (56, 57). Most genetic abnormalities are difficult to verify at the level of variation, however significance is repeatedly observed at the gene and pathway levels. (58).

In this study, we compared the transcriptome signatures of CC and PFC across ASD-affected individuals versus healthy controls. WGCNA was performed to identify co-expression patterns of DEGs and their protein–protein interactors. Then, based on DVMT and SFARI genes, 12 of 49 (6/24 for CC set, 6/25 for PFC set) dysfunctional modules significantly enriched for genomic variants and/or known ASD susceptibility genes were identified as ASD-correlated modules and kept for additional analysis. GO-BP and KEGG pathway enrichment analysis showed that pathophysiological process of both CC and PFC in ASD seem to converge on specific molecular dysregulation, mainly for synaptic and neuronal signaling pathways, which in line with earlier studies for *de novo* mutations associated with ASD (59–61). In addition, PFC M5, which showed significantly enriched for DVMT but not SFARI genes, shared most ASD-related GO terms and pathways with CC M4 and M6, supporting its potential role in the disorder. Moreover, we identified pivot regulators that might perturb gene regulation or affect gene function by constructing multi factor-mediated regulation subnetworks.

Cross-talk between dysfunctional modules revealed that modules can not only affect each other through PPIs but be perturbed directly or indirectly by multi-regulators. Remarkably, we found that there were significantly more interactions between CC-M4, -M6 and PFC-M2. These three modules mainly converged on synaptic and neuronal developmental functions indicated the close correlation between different brain area, which also highlighted the importance of integrative strategy to ASD. Collectively, our system-level analysis of the ASD brain transcriptome demonstrated the existence of common and distinct molecular abnormalities in CC and PFC for the first time, and implicated different distribution of genomic variants burden as underlying mechanisms of neuronal dysfunction for the disorder.

There are also limitations to this study. First, our analysis is biased by the available data. Gene transcriptional profiling in ASD is mainly performed on post-mortem brain samples, but the availability of human ASD brain tissues often represents a significant challenge. Given this fact, we can only use tissues from different individuals as sample sources, which may reduce the comparability to some extent. Second, the SFARI and DVMT gene list, although comprehensive, is likely to have potential curation bias, further confirmations are needed. Third, gene expression studies highlight just one aspect of gene regulation. We have considered some additional regulatory levels (TFs, miRNAs, and lncRNAs), nevertheless, post-translational regulation mechanisms that affect the development of the disease are still under investigation.

## Data Availability Statement

Gene expression profile GSE62098 and GSE102741 were downloaded from the NCBI Gene Expression Omnibus (GEO).

## Author Contributions

XL and YZ contributed to data analysis and wrote the manuscript. LWa, YL, and YH contributed to plot pictures and tables. ZG, CS and XZ contributed to data collection. DW and SL were responsible for the study design and SL revised the manuscript. LWu supervised the whole study.

## Funding

This study was funded by grants from the National Natural Science Foundation of China (81973068), the China Postdoctoral Science Foundation (2014M561375), and the Scientific Research Fund of Heilongjiang Postdoctoral Program (LBH-Z14153).

## Conflict of Interest

The authors declare that the research was conducted in the absence of any commercial or financial relationships that could be construed as a potential conflict of interest.
