# Supplementary figures and images for: Integrated Analysis of Brain Transcriptome Reveals Convergent Molecular Pathways in Autism Spectrum Disorder
Source: Front Psychiatry. 2019 Oct 8;10:706. doi: 10.3389/fpsyt.2019.00706 (PMC6795181; doi:10.3389/fpsyt.2019.00706)

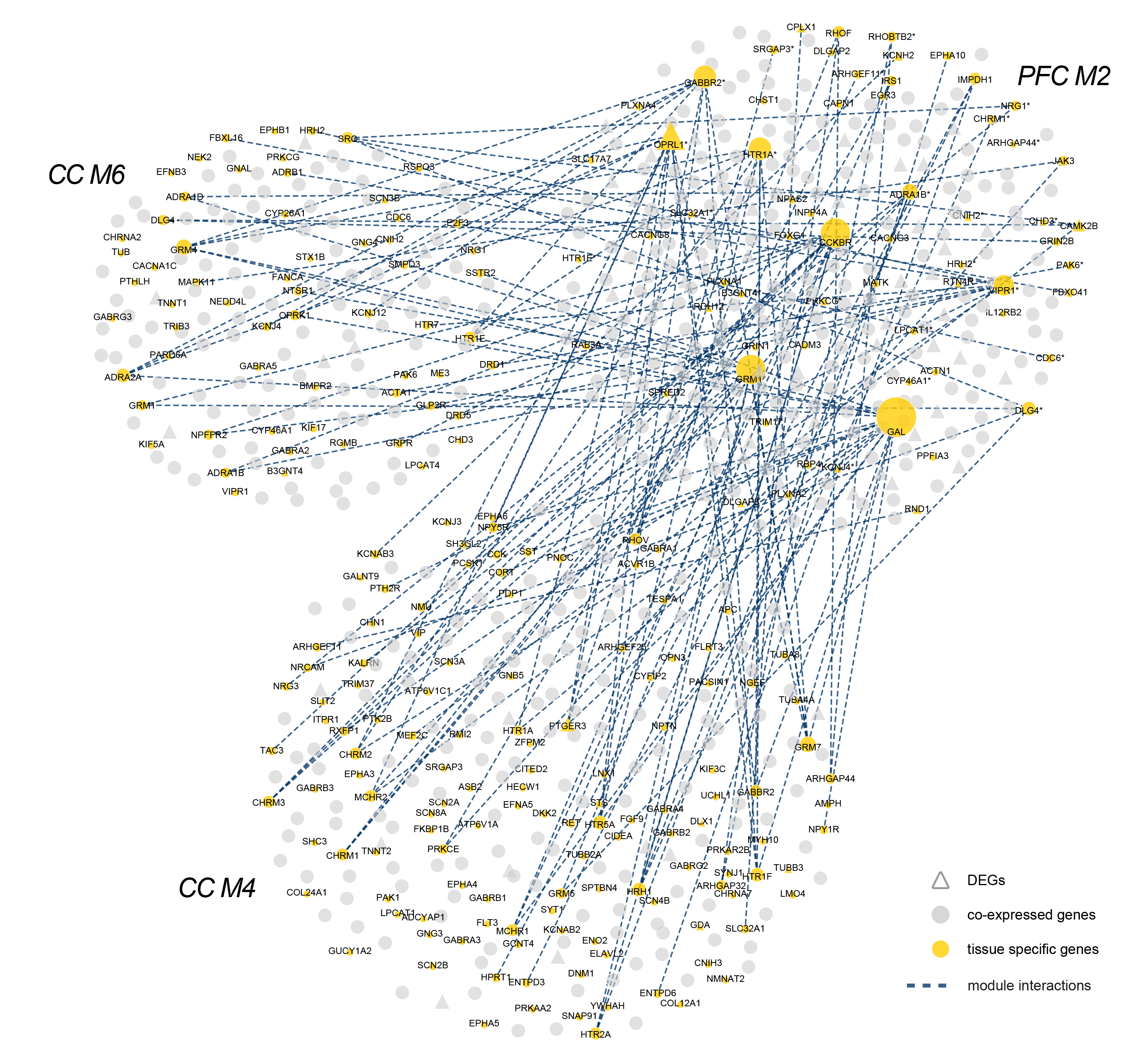

Supplement: Supplementary file 6 [file Image_1.tif]
